# Cemiplimab and diabetic ketoacidosis: a case report of a rare endocrinopathy associated with immune checkpoint inhibitors

**DOI:** 10.3389/fendo.2025.1550702

**Published:** 2025-03-25

**Authors:** Anna Arecco, Cristian Petolicchio, Alessandro Pastorino, Enrica Teresa Tanda, Lara Vera, Mara Boschetti, Francesco Cocchiara, Davide Carlo Maggi, Diego Ferone, Federico Gatto

**Affiliations:** ^1^ Endocrinology Unit, Department of Internal Medicine and Medical Specialties, School of Medical and Pharmaceutical Sciences, University of Genova, Genova, Italy; ^2^ Medical Oncology Unit 1, IRCCS Ospedale Policlinico San Martino, Genova, Italy; ^3^ Department of Internal Medicine and Medical Specialties, University of Genova, Genova, Italy; ^4^ Medical Oncology 2, IRCCS Policlinico San Martino, Genova, Italy; ^5^ Endocrinology Unit, IRCCS Ospedale Policlinico San Martino, Genova, Italy

**Keywords:** immune checkpoint inhibitors, cemiplimab, diabetic ketoacidosis, immune-related adverse events, diabetes mellitus, anti-PD-1 monoclonal antibody, ICI-induced hepatotoxicity, squamous cell carcinoma

## Abstract

**Background:**

Immune checkpoint inhibitors (ICIs) have revolutionised the cancer treatment landscape in the last decades, improving the outcome of several tumours, such as cutaneous squamous cell carcinoma (cSCC). ICIs are antibodies blocking several immune checkpoint pathways, as cytotoxic T lymphocyte-associated antigen 4 (CTLA-4) and programmed cell death 1 (PD-1) with its ligand PD-L1. However, the activation of immune response can cause a broad range of side effects, called immune-related adverse events (irAEs). Endocrine irAEs are mainly represented by thyroid dysfunctions (thyrotoxicosis or hypothyroidism) and hypophysitis, while adrenal insufficiency and diabetes mellitus (DM) are less common. Diabetic ketoacidosis (DKA) is a potential life-threatening presentation of ICI-induced insulin-dependent DM (IDDM). This report presents a rare case of DKA and IDDM secondary to anti-PD-1 antibody cemiplimab therapy, and this is the third described in the literature to date.

**Case presentation:**

We describe the case of a 62-year-old female patient with metastatic perianal squamous cell carcinoma who developed DKA and IDDM after the fifth cycle of cemiplimab. Hyperglycemia (1187 mg/dL), metabolic acidosis (pH 7.27) with bicarbonate levels of 11.9 mmol/L, arterial partial pressure of carbon dioxide of 25.7 mmHg with increased anion gap (equal to 25), and hyperketonuria were present. Adequate glycaemic control was difficult to maintain, and intravenously therapy (insulin, sodium bicarbonate, potassium, and fluids) was required for a long time. Subcutaneous basal-bolus insulin treatment was started, but glycaemic control was scarce, also due to the concomitant administration of prednisone for immune-related hepatotoxicity, until the subject’s death.

**Conclusion:**

This report underlines the importance of the awareness on endocrine irAEs with ICIs, particularly life-threatening DKA. A baseline assessment of glycemia and glycated hemoglobin is mandatory, and we recommend a close monitoring of glycemic trend over time during ICIs therapy. Patients and their caregivers should be informed and counselled to recognise DKA signs and symptoms.

## 1 Introduction

Tumour immunotherapy, including immune checkpoint inhibitors (ICIs) and adoptive cell therapy, has radically changed the cancer treatment landscape, affording long-term benefits in metastatic patients and increasing the chances of cure in the adjuvant setting (1). Immune checkpoints are small molecules that play a crucial role in maintaining immune homeostasis and tolerance, thus modulating the duration and amplitude of the immune response. ICIs disrupt inhibitory signals from neoplastic cells to immune effector cells, allowing activated T-cells to target the neoplastic cells (2). They are antibodies blocking several immune checkpoint pathways, as cytotoxic T lymphocyte-associated antigen 4 (CTLA-4) and programmed cell death 1 (PD-1) with its ligand PD-L1 (3).

Along with ICIs such as nivolumab and pembrolizumab, cemiplimab is a human recombinant monoclonal immunoglobulin G subclass 4 (IgG4) antibody targeted to the PD-1 receptor, first approved by the Food and Drug Administration (FDA) in 2018 and by the European Medicines Agency (EMA) in 2019 for locally advanced or metastatic cutaneous squamous cell carcinoma (NCT02760498) (4, 5). It has demonstrated efficacy with response rates of 47–50% (4). As stated, the PD-1 receptor is an immune checkpoint molecule expressed on effector T, B and NK cells. One of its ligands is PD-L1, expressed in various types of self-cells (as tubular epithelial, endothelial cells, fibroblastic reticular cells, pancreatic islet cells, astrocytes, and neurons), but it is often present also in tumour cells as a biological escape mechanism. Indeed, activation of the PD-1 receptor by PD-L1 down-regulates T cell responses, avoiding autoimmunity and host organ injury. The binding of anti-PD-1 antibody to the PD-1 receptor prevents PD-L1 action and activation of the programmed cell death pathways, resulting in the continued activation and proliferation of T cells against tumour cells (6).

Treatment with ICIs improved the outcome of several solid tumours, such as melanoma, breast cancer, non-small cell lung carcinoma, renal cell carcinoma, colorectal cancer and endometrial cancer (7–10). However, the immune response activation can cause specific side effects called immune-related adverse events (irAEs) (6). Almost all organs can be affected by irAEs, such as skin, gastrointestinal tract and liver, followed by endocrine organs, nervous system, lungs, heart, joints, pancreas and kidneys (11). Endocrine irAEs are mainly represented by thyroid dysfunctions (thyrotoxicosis or hypothyroidism) and hypophysitis, while adrenal insufficiency and diabetes mellitus (DM) are less common (12).

The incidence of ICI-induced DM ranges from 0.9 to 2%, and diabetic ketoacidosis (DKA) is reported in up to 70% of cases with DM at diagnosis (12). DKA is a life-threatening endocrine emergency along with hyperosmolar hyperglycaemic syndrome (13). According to the American Diabetes Association (ADA), DKA is defined by the triad of hyperglycaemia (blood glucose greater than 13.9 mmol/L or 250 mg/dL), anion gap metabolic acidosis [arterial pH less than or equal to 7.3, serum bicarbonate less than or equal to 18 mmol/L (mEq/L), blood anion gap greater than 10 mmol/L (mEq/L)] and ketosis (positive serum or urine ketone on a semi-quantitative test) (14). DKA most commonly occurs in type 1 diabetes mellitus (T1D) but may occur in subjects with type 2 diabetes mellitus (T2D) (13). It is caused by an absolute or relative insulin deficiency in T1D or a relative insulin deficiency associated with insulin resistance in T2D. Different conditions can precipitate the development of hyperglycemia and subsequent ketoacidosis, such as infections, non-adherence to insulin therapy, acute major illnesses (i.e. myocardial infarction, sepsis, pancreatitis), stress and trauma (15). Also, the use of certain medications, such as glucocorticoids (16), atypical antipsychotic agents (17), sodium-glucose co-transporter-2 (SGLT-2) inhibitors (18) or ICIs (19), may lead to DKA.

Cutaneous squamous cell carcinoma (cSCC) accounts for 20% of skin cancers (20). It often presents as a scaly, red, or bleeding lesion typically on sun-exposed areas. Diagnosis is made by skin biopsy; if needed, enhanced computed tomography (CT) may help to evaluate lymph nodes, soft tissue, or bone involvement. Surgery is the first-line treatment, while radiotherapy can be used as adjuvant therapy in case of perineural invasion or if the patient is not a surgical candidate. Until a few years ago, systemic therapies for advanced cSCC included platinum-based chemotherapies, capecitabine, and epidermal growth factor receptor inhibitors, such as cetuximab (21); however, none of these therapies provided long-term responses and the few benefits obtained were lost quickly. Recently, ICIs were approved for use in locally advanced and metastatic cSCC (4). This disease is characterized by a poor quality of life, and the advent of immunotherapy has afforded a high rate of objective responses which, in addition to increasing progression-free survival and overall survival, radically improve the quality of life of patients (22). Furthermore, immunotherapy replaces chemotherapy and radiotherapy in the metastatic disease, offering a better tolerability profile.

Herein, we describe the case of a female subject with a metastatic perianal squamous cell carcinoma (SCC) who developed DKA and insulin-dependent DM (IDDM) after the fifth cycle of cemiplimab. To date, only two case reports of DKA and IDDM in patients treated with cemiplimab are described (23, 24). This report underlines the need for baseline assessment of glycemia and glycated hemoglobin (HbA_1c_) and close monitoring of glycemic trend over time, which is recommended in candidates for cemiplimab therapy.

## 2 Case description

In March 2022, a 62-year-old Caucasian female was admitted to our hospital for altered mental status and vomiting. As concerns the clinical history, a diagnosis of vulvar lichen planus was made in 2017, unsuccessfully treated with platelet-rich plasma injections, and therefore a vulvoplasty was performed. Other patient co-morbidities included Hashimoto’s thyroiditis, osteoporosis, arterial hypertension well controlled with angiotensin-converting enzyme inhibitor, primary amenorrhea (unknown cause), and alopecia. She took combined estrogen-progestogen replacement therapy for 15 years, discontinued at the age of 35. There was no personal or family history of pancreatitis, diabetes (also gestational diabetes) or obesity. In November 2021, a large painful ulcerated perianal lesion extended to the gluteal region and to the vulva anteriorly associated with anal stenosis was found. The histological examination of the lesion led to the diagnosis of a moderately differentiated cutaneous SCC. The abdominal magnetic resonance imaging (MRI) performed at baseline showed the infiltration of the rectal fascia, which appeared thickened, and inflammation of the external anal sphincter. To define the best therapeutic work-up, a multidisciplinary discussion was started, leading to the choice of therapy with cemiplimab, an anti-PD-1 monoclonal antibody administered at the dose of 350 mg intravenously every three weeks, with complete resolution of the painful symptoms and partial response of the lesion after two cycles.

Before cemiplimab therapy started, the patient reported a weight loss of around 7 kg in the previous 6 months: her body weight was 42.5 kg for a height of 1.58 m with a body mass index (BMI) of 17 kg/m^2^. On blood tests, fasting blood glucose was 86 mg/dL with a HbA_1c_ value of 36 mmol/L (5.5%) with haemoglobin 12.9 g/dL. Her liver function tests (LFTs) were normal: aspartate aminotransferase (AST; 18 UI/L [0.45 xULN]), alanine aminotransferase (ALT; 21 U/L [0.52 xULN]) and gamma-glutamyl transpeptidase (γGT; 8 U/L [0.16 xULN]).

**Figure 1:**
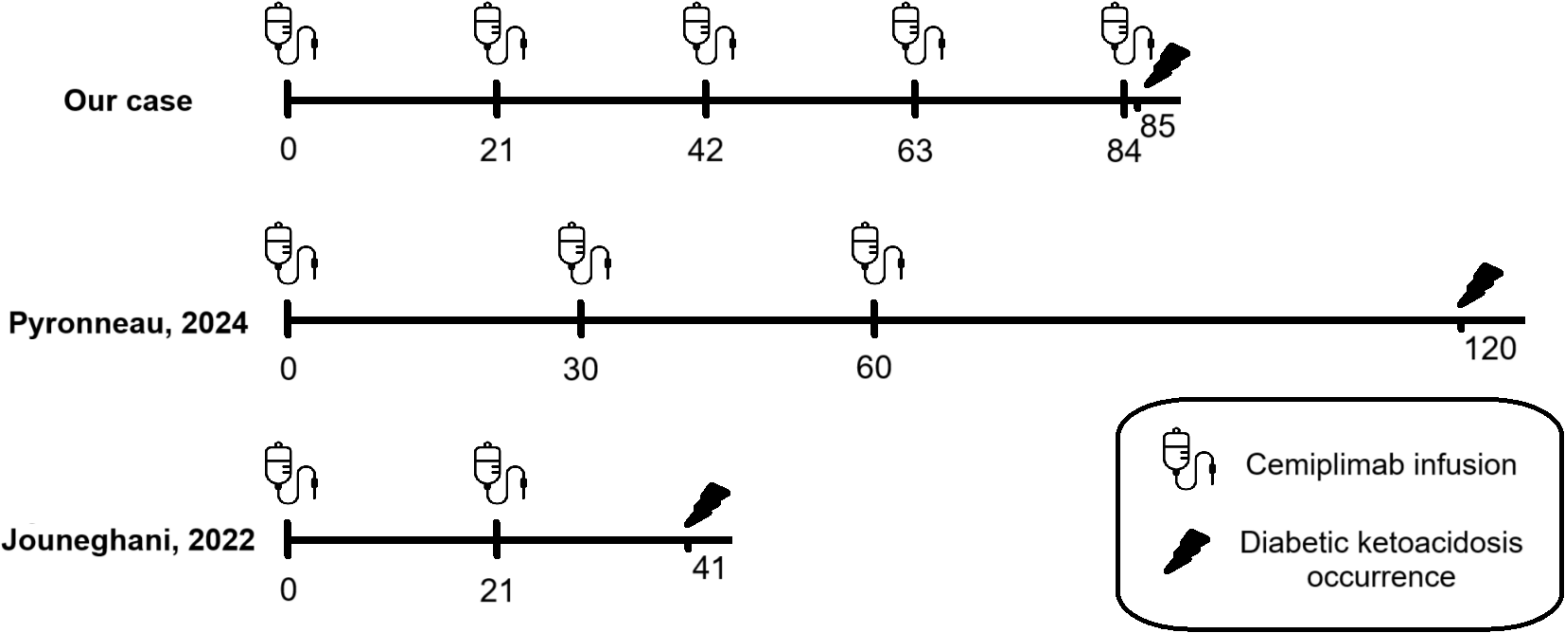
Comparison timeline between case reports about DKA secondary to cemiplimab therapy published in the literature to date: development of DKA is different: one day after the fifth cycle of cemiplimab in our patient, after two months of ICI’s discontinuation after two cycles in patient described by Pyronneau and twenty days after the second cycle in patient described by Jouneghani.

The day after the fifth cemiplimab infusion (**Figure 1**), the patient was admitted to the emergency department for asthenia, vomiting, and lethargy. Physical examination revealed drowsiness and dry mucosa, tachycardia, and tachypnea; no fever or blood pressure alteration were found. Arterial blood gas analysis revealed metabolic acidosis (pH 6.8) with extremely low bicarbonate levels (HCO_3_^-^; 2.7 mmol/L) and low arterial partial pressure of carbon dioxide (PaCO_2_; 15.0 mmHg). Moderate-high potassium serum levels (5.5 mmol/L) and low sodium serum levels (123 mmol/L) were found. Glucose values were undetectable and a mild elevation of lactic acid (2.6 mmol/L) was detected. Therefore, treatment with intravenous insulin, sodium bicarbonate, potassium and fluids were immediately started. Blood tests revealed severe hyperglycemia (1187 mg/dL) and confirmed metabolic acidosis (pH 7.27) with HCO_3_^-^ levels of 11.9 mmol/L, PaCO_2_ 25.7 mmHg, characterized by an increased anion gap (equal to 25); a remarkable hyperketonuria was detected.

A Naranjo nomogram with a score of 5 (range 0-13) indicated a probable relationship between cemiplimab and IDDM (**Table 1**); therefore, cemiplimab therapy was discontinued.

**Table 1:** The Naranjo adverse drug reaction (ADR) probability scale questionnaire.

To assess the adverse drug reaction, please answer the following questionnaire and give the pertinent score				
	Yes	No	Do not know	Score
1. Are there previous conclusive reports on this reaction?	+1	0	0	+1
2. Did the adverse event occur after the suspected drug was administered?	+2	-1	0	+2
3. Did the adverse reaction improve when the drug was discontinued or a specific antagonist was administered?	+1	0	0	0
4. Did the adverse reaction reappear when the drug was readministered?	+2	- 1	0	-1
5. Are there alternative causes (other than the drug) that could have on their own caused the reaction?	-1	+2	0	+2
6. Did the reaction reappear when a placebo was given?	-1	+1	0	0
7. Was the drug detected in the blood (or other fluids) in concentrations known to be toxic?	+1	0	0	0
8. Was the reaction more severe when the dose was increased or less severe when the dose was decreased?	+1	0	0	0
9. Did the patient have a similar reaction to the same or similar drugs in any previous exposure?	+1	0	0	0
10. Was the adverse event confirmed by any objective evidence?	+1	0	0	+1
Total score				5

Adapted from Naranjo CA, Busto U, Sellers EM, et al. A method for estimating the probability of adverse drug reactions. *Clin Pharmacol Ther.* 1981;30 (2):239-245. Scoring: ≥ 9 = definite ADR; 5-8 = probable ADR; 1-4 = possible ADR; 0 = doubtful ADR.

During hospitalization, the inadequate glycaemic control required persistent intravenous therapy with insulin, sodium bicarbonate, potassium, and fluids for more than two weeks. A following improvement of the clinical picture allowed the shift to subcutaneous administration of insulin, with a basal-bolus scheme at a medium daily dosage of 0.4 U/Kg. Blood sampling revealed a HbA_1c_ of 52 mmol/mol (6.9%) with haemoglobin 12.1 g/dL, undetectable level C-peptide levels (both after eight hours fasting and on random sampling), and negative autoimmunity for anti-GAD (glutamic acid decarboxylase) and anti-insulin antibodies.

After two weeks of hospitalization, a significant increase of the liver enzymes AST (191 U/L [4.78 xULN]) and ALT (308 U/L [7.7 xULN]) was detected. Gamma-glutamyl transpeptidase (γGT) was within the normal range (22 U/L, 0.44 xULN). An evaluation for hepatotropic virus infection was carried out, although revealing only HbsAb (antibody of hepatitis B surface) positivity, suggestive for a previous immunization against hepatitis B virus. No signs of biliary sludge or cholestasis were detected at abdominal ultrasound, and no hepatotoxic drugs were recently administered. Antinuclear antibodies (ANA) were negative, while anti-smooth muscle antibody (ASMA), liver kidney microsome type 1 (anti-LKM-1) antibodies and antibodies against soluble liver antigen/liver-pancreas (SLA/LP) were not performed. A grade 3 immune-related hepatotoxicity was suspected, and prednisone 50 mg once a day was started, with consequent improvement of biochemical parameters (AST 26 UI/L [0.65 xULN], ALT 39 U/L [0.99 xULN], γGT 21 U/L [0.42 xULN]) within two weeks, so prednisone was reduced to 25 mg/day. However, the glycemic profile worsened, therefore the basal-bolus insulin therapy was increased to a medium daily dosage of 0.48 U/Kg, and a continuous glucose monitoring was started. Though the increase in medium daily insulin dose and reduction of prednisone to 25 mg/day after one week, it was hard to maintain adequate blood glucose control.

Since at this stage an autoimmune polyglandular syndrome was suspected, anti-ovarian, anti-adrenal, anti-endomysial, and extractable nuclear antigens (ENA) antibodies were assayed, but they all tested negative.

During hospitalization, elevated thyroid stimulating hormone (TSH, 16.82 mUI/L) with normal free thyroid hormones [free triiodothyronine (fT3) 1.09 ng/L, free thyroxine (fT4) 9.39 ng/L)] were found, despite the ongoing treatment for Hashimoto’s thyroiditis. Before starting the immune checkpoint inhibitor and before every treatment cycle, TSH was within the normal range with levothyroxine therapy. In suspicion of a worsening of the known thyroid dysfunction due to ICI treatment, levothyroxine was increased to 75 mcg daily.

Other pituitary hormones than TSH were evaluated during the hospitalization: the corticotrope axis was suppressed due to concomitant corticosteroid therapy; lactotroph and somatotroph axes were preserved with prolactin (PRL) 8.48 µg/L and insulin-like growth factor-1 (IGF-1) 66 µg/L; gonadotroph axis revealed a deficit, since luteinizing hormone (LH) was 2.37 U/L, and follicle-stimulating hormone (FSH) was 4.36 U/L (inappropriately low for the patient’s age). A brain CT detected a primary empty sella, but a contrast-enhanced pituitary MRI was not performed to further explore this finding.

Forty days after the onset of DKA, a tumor disease progression (increased size of the perianal-anal lesion) was noted, so after another multidisciplinary discussion, cemiplimab therapy was restarted.

After six cycles of cemiplimab, a new episode of elevation of liver enzymes (AST 181 U/L [4.52 xULN] and ALT 194 U/L [4.85 xULN]) and gamma-glutamyl transpeptidase (γGT 628 U/L [12.56 xULN]) occurred, so corticosteroid therapy with prednisone 50 mg daily was restarted. Despite treatment with prednisone, LFTs worsened (AST 758 U/L [18.95 xULN], ALT 527 U/L [13.18 xULN], γGT 1540 U/L [30.8 xULN]), leading to the initiation of intravenous methylprednisone at a dosage of 2 mg/kg daily. Methylprednisone was gradually reduced and, after ten days, switched to prednisone 25 mg/day with improvements in liver function tests. The glycemic profile worsened, prompting an increase in basal-bolus insulin therapy to a medium daily dosage of 1.35 U/Kg, alongside continued glycemic monitoring. Cemiplimab was permanently discontinued due to a lack of clinical improvement and the occurrence of a new episode of grade 3 immune-related hepatotoxicity.

From September 2022 chemotherapy with 5-fluorouracil and mitomycin associated with local radiotherapy for SCC was started with disease stability. Due to the poor performance status, salvage surgery was not performed, and from March 2023, chemotherapy with carboplatin and paclitaxel was started. Disease progression occurred, and the patient died of septic shock secondary to *Staphylococcus hominis* and *Staphylococcus epidermidis* infection in August 2023.

**Table 2** shows the subject’s characteristics with ICI-associated DKA.

**Table 2:** Characteristics of reported patient with ICI-associated DKA.

		Reference values
Age (year)	62	
Sex	Female	
Underlying cancer	Squamous cell carcinoma	
Type of ICI	Cemiplimab	
Previous history of DM	No	
HbA_1c_ (%)	6.9	4.3-5.7
C-peptide (ng/mL)	<0.0	0.8-4.2
Glucose (mg/dL)	1187	65-110
Serum pH	6.8	7.35-7.45
pCO_2_ (mmHg)	15.0	23.0-27.0
Serum bicarbonate (mmol/L)	2.7	22.0-25.0
Urine ketone	++++	Absent
Time to diagnosis after starting ICI		
Number of doses	5	
Onset in weeks	12	
Beta-cell autoantibodies		
GAD Ab (U/mL)	0.78	0.00-10.0
Anti-insula Ab (U/mL)	0.1	0.0-1.1
Other endocrinopathies		
Thyroid dysfuntion	Yes	
Adrenal insuffiency	Not valuable	
Tumour response	Progressive disease	
ICI therapy after DKA	Stop	

*ICI*, immune checkpoint inhibitor; *DM*, diabetes mellitus; HbA_1c_, glycated haemoglobin; *GAD*, glutamic acid decarboxylase; *Ab*, antibody; *DKA*, diabetic ketoacidosis.

## 3 Discussion

This case report is an interesting example of the challenges met by clinicians in the management of immune-related adverse events secondary to ICIs, particularly in the case of ICI-related DM and its possible presentation as diabetic ketoacidosis.

DM is a rare irAE after ICI treatment, with an overall incidence between 0.9 to 2% (12). A female-to-male ratio ranges from 1:1.2 to 1:9, and the mean age at onset is over 60 years. Up to 76% of patients developing ICI-induced DM received anti-PD-1, 8% anti-PD-L1, while only 4% received anti-CTLA-4 antibodies. Up to 70% of ICI-induced DM cases present as DKA, which can occur from 7 to 25 weeks after treatment initiation (25). With anti-PD1 inhibitors, several case reports have reported new-onset DM following nivolumab and pembrolizumab treatment (26–28), but only two cases after cemiplimab treatment are described to date (23, 24).

As reported in **Table 3**, our patient is a Caucasian female diagnosed with squamous cell carcinoma who received cemiplimab therapy and had no prior history of diabetes who developed a severe diabetic ketoacidosis 12 weeks after starting cemiplimab therapy. She had not undergone previous treatment with other ICIs, unlike the patients in Pyronneau’s case report, who had received prior treatment with pembrolizumab. Furthermore, our patient is younger (62 years) than those described by Pyronneau and Jouneghani (77 and 74 years, respectively). She presented with metabolic acidosis characterised by an elevated anion gap, along with glycosuria and ketonuria, and her HbA_1c_ value at irAE presentation (6.9%) was lower than those reported by Pyronneau and Jouneghani (8.3% and 8.4%, respectively). Anti-GAD antibodies were negative, as reported by Pyronneau.

**Table 3:** Comparison between patients with ICI-associated DKA.

	Our case	Pyronneau, 2024 (24)	Jouneghani, 2022 (23)
Age (year)	62	77	74
Sex	Female	Female	Female
Ethnicity	Caucasian	Caucasian	Caucasian
Underlying cancer	Squamous cell carcinoma	Squamous cell carcinoma	Squamous cell carcinoma
Type of ICI	Cemiplimab	Cemiplimab	Cemiplimab
Previous other ICI	No	Pembrolizumab	NA
Previous history of DM	No	NA	No
Biochemical exams at the time of irAE presentation			
HbA_1c_ (%)	6.9	8.3	8.4
C-peptide (ng/mL)	<0.0	NA	0.2
Glucose (mg/dL)	1187	1186	623
β-hydroxybutyrate (mg/dL)	Not valuable	Elevated	Elevated
Serum pH	6.8	NA	Acidosis
Serum bicarbonate (mmol/L)	2.7	15	NA
Anion gap (mmol/L)	Elevated (25)	Elevated (27)	Elevated
Creatinine (mg/dL)	1.6	2.5	NA
Lactic acid (mmol/L)	2.6	5.4	NA
Urine glucose	Positive	Positive	NA
Urine ketone	Positive	Positive	NA
Time to diagnosis after starting ICI			
Number of doses	5	3	2
Onset in weeks	12	16	6
Beta-cell autoantibodies			
GAD Ab (U/mL)	Negative	Negative	Positive

*ICI*, immune checkpoint inhibitor; *DM*, diabetes mellitus; HbA_1c_, glycated haemoglobin; *GAD*, glutamic acid decarboxylase; *Ab*, antibody; *NA*, not available; *irAE*, immune-related adverse event.

A relevant proportion of patients with ICI-induced DM have elevated HbA_1c_ levels (>7.5%), low or undetectable C-peptide levels, and at least one positive DM-specific antibody at diagnosis (most commonly anti-GAD antibodies) (26, 29, 30). Similarly to T1D, an inappropriate hyperactivation of the immune system secondary to alteration in the PD-1/PD-L1 or CTLA-4 pathways may cause severe impairment and dysfunction of insulin-producing β-cells. To date, the mechanisms underlying ICI-T1D remain unclear. A T-cell-mediated insulitis is a possible mechanism hypothesised: after ICI treatment, PD-L1 molecules, expressed on the pancreatic β-cells, cannot bind the PD-1 receptor on autoreactive T cells (31). Therefore, PD-1/PDL1 inhibition induces pancreatic β-cell damage through activated autoreactive T-cells by releasing interferons and nitric oxide, which activate monocyte-derived macrophages, leading to insulin deficiency and low/undetectable C-peptide levels (32). Particularly, CD8+ cytotoxic T-cells, producing interferon-γ (IFN-γ) and tumor necrosis factor α (TNF-α), infiltrate the pancreatic β-cells (33) resulting in the possible direct cytotoxic killing of β-cells via the action of perforin-granzymes, as observed in non-obese diabetic mouse models (34). As some studies have reported that around 40% of cases are positive for islet-related autoantibodies (35), antibody-mediated β-cell damage is another possible mechanism. The relationship between islet-related autoantibodies and pathological conditions remains unclear, and further investigations are required (36).

A recent study analysed with immunohistochemistry pancreatic specimens from three individuals with ICI-related T1D, three patients who had received ICI therapy but did not develop T1D (non-T1D), and seven people without T1D who did not receive ICIs (controls) (37). In ICI-related T1D, the β-cell area decreased, and the α-cell area increased compared with non-T1D and control subjects. The number of CD3+ cells, primarily CD8+ cells, around the islets increased in ICI-related T1D and non-T1D subjects compared to control subjects. Meanwhile, the number of CD68+ cells, identified as macrophages, around the islets increased in ICI-related T1D compared to non-T1D and control subjects. Furthermore, in ICI-related T1D and non-T1D patients, CD8+ lymphocytes predominated over CD4+ lymphocytes, indicating T cell-mediated cytotoxicity. The expression ratios of PD-L1 on islets decreased in non-T1D cases and nearly disappeared in ICI-related T1D, unlike in controls. According to the authors, the absence of PD-L1 expression on β-cells and infiltration of macrophages and T cells around the islets may be responsible for ICI-related T1D onset (37).

Different scientific societies, such as the American Diabetes Association (ADA), the European Society for Medical Oncology (ESMO), the European Society of Endocrinology (ESE) and the American Society of Clinical Oncology (ASCO), have proposed clinical practice guidelines or practical recommendations for managing ICI-induced DM or DKA (12, 38–40). A recent review has been published on the pathophysiology, diagnosis, and management of ICI-induced DM (41).

Regular monitoring of blood glucose levels is essential for the early diagnosis of ICI-induced DM and for preventing DKA (12, 39, 41). While there is no specific timeline or target ranges for blood glucose assessments, various organisations, including the National Comprehensive Cancer Network (NCCN) and the Japan Endocrine Society, recommend evaluating blood glucose regularly at each treatment cycle or visit (42, 43). Furthermore, patients receiving ICIs, particularly anti-PD1 or PD-L1 treatments, should be educated about the acute symptoms of diabetes, such as polyuria, polydipsia, and weight loss, as well as the symptoms of ketoacidosis, including nausea, vomiting, and gastrointestinal disorders (44, 45). Our patient was carefully educated about the symptoms to be recognised, and her blood glucose levels were assessed before each cemiplimab infusion. No alteration was detected in blood tests before the onset of DKA.

Management of ICI therapy differs based on the blood glucose values and the severity of typical symptoms reported (39, 40):

- If the patient has no or mild symptoms, with fasting glucose levels ranging from 126 mg/dL to 160 mg/dL (grade 1), ICI therapy can be continued with close clinical follow-up and laboratory evaluation.

- If the patient has moderate symptoms and fasting glucose levels greater than 160 mg/dL (up to 250 mg/dL, according to ASCO guidelines) (grade 2), ICIs may be temporarily suspended until symptom resolution and glucose control are achieved. Close follow-up of pH, urine ketones and blood glucose levels, together with endocrine consultation, are recommended, along with initiating insulin therapy in case of persistent hyperglycemia. The usual dosage for insulin ranges from 0.3 to 0.4 units per kilogram of body weight daily, with half of the dosage administered as long-acting insulin and the remaining half as rapid-acting insulin at mealtimes. Insulin dosage will be adjusted according to blood glucose trends.

- If the patient experiences severe symptoms (with fasting glucose between >250 and 500 mg/dL according to ASCO guidelines) (grade 3) or life-threatening symptoms (with fasting glucose > 500 mg/dL, ketoacidosis, or other metabolic abnormalities according to ASCO guidelines), it is recommended suspending ICI until glucose control is achieved with a reduction of toxicity to ≤ grade 1. Endocrine consultation is recommended for all patients, and hospitalisation may be necessary, particularly in cases of DKA. It is advisable to initiate insulin therapy immediately. If DKA is diagnosed, it is recommended to refer to guidelines for DKA treatment (14, 46).

Insulin is the treatment cornerstone of ICI-induced DM: in case of DKA, intravenous hydration, insulin therapy, and the restoration of electrolyte balance are mandatory (12). In almost all cases, insulin treatment in ICI-induced DM is lifelong: to date, only four cases in which insulin therapy have been stopped are described (47–50). Unlike other irAEs, there is no evidence that high-dose glucocorticoids may improve DM or its management, while it is well-known that glucocorticoids may increase serum glucose levels. Our patient needed insulin therapy until death. When prednisone was administered to treat liver function tests (LFTs) elevation, worsening of glycaemic control and need for increased insulin dosage were observed.

Establishing biomarkers is important for identifying patients at risk of developing ICI-related DM or DKA before initiating treatment (51). A case series reported that the prevalence of HLA-DR4 was significantly higher in patients with ICI-induced DM (76%) than in U.S. Caucasians (17.3%) or even in patients with spontaneous type 1 DM (30). Other HLA susceptibility alleles (HLA-A2, HLA-DR3, HLA-DQ8) were not significantly higher or were of the same frequency in the U.S. general population (30). Additionally, positive islet autoantibodies at baseline, such as anti-GAD, anti-islet antigen 2 (IA2), anti-zinc transporter 8 (ZnT8), and islet cell antibodies, may serve as biomarkers for the development of ICI-induced DM. As reported by Stamatouli et al., in 27 patients who developed ICI-induced T1D, the prevalence of anti-GAD, anti-IA2, anti-ZnT8, and islet cell antibodies was 36% (9/25 patients), 21% (5/24 patients), 10% (2/20 patients), and 11% (2/19 patients), respectively (30). Our patient had undetectable C-peptide levels and negative DM autoimmunity (with the limitation that only anti-GAD and anti-insulin antibodies were assayed at our laboratory), as reported elsewhere (52). No sequencing of HLA alleles was performed.

DKA is linked to higher rates of morbidity, mortality, and healthcare costs (53). Recent estimates indicate that inpatient mortality rates during hospitalization for DKA range from 0.20% in individuals with T1D to 1.04% in those with T2D (54). Furthermore, individuals discharged after experiencing DKA have a one-year age-adjusted mortality rate that is 13 times higher than that of the general population (55). According to our knowledge, mortality secondary to ICI-induced DKA has not been assessed to date. People with diabetes have a 1.5- to 4-fold increased risk of infections, such as pyelonephritis, osteomyelitis, foot infection, pneumonia, skin infections, and sepsis (56). Our patient died one year and five months after the DKA episode due to septic shock; during hospitalization, maintaining adequate glycaemic control was challenging. The patient’s poor general clinical condition, due to the rapid progression of advanced cSCC, combined with poor glycaemic control, significantly contributed to the negative outcome. There is no evidence that intensive blood-glucose-lowering decreases infection rates in people with diabetes (57). A useful tool to prevent infections is vaccinations (COVID-19, hepatitis B, influenza, pneumococcal, tetanus, diphtheria, acellular pertussis and zoster in patients ≥ with 50 years) as recommended by the latest ADA guidelines (58).

A history of Hashimoto’s thyroiditis with thyroperoxidase antibody positivity required the evaluation of other associated autoimmune diseases as a part of a (suspect) polyglandular autoimmune syndrome (PAS). PAS is defined by the coexistence of at least two immune-mediated endocrinopathies, and different types are identified (59). In our patient, no signs or symptoms of hypoparathyroidism or Addison’s disease were present, as well as no gastrointestinal, rheumatological, dermatological, haematological, or neurological autoimmune diseases were detected. Her clinical history of primary amenorrhea gave rise to the suspicion of a primary ovarian insufficiency, but no additional clinical information could be retrieved, and anti-ovarian antibodies were negative.

This clinical case is also of interest since the patient was suspected for another endocrine irAE, namely hypothyroidism. Thyroid dysfunction represents the most common endocrine irAE: hypothyroidism has an estimated prevalence of 2.5-3.8% with anti-CTLA4 antibodies, 3.9–8.5% with anti-PD1/PDL1 antibodies and 10.2-16.4% with combination treatment. ICI-induced hypothyroidism is mainly due to a direct thyroid damage, while central hypothyroidism, secondary to hypophysitis, is rare (12). Controlled hypothyroidism is not a contraindication for the initiation of ICI therapy *per se*, but the patient needs to be carefully monitored, since it may need higher doses of levothyroxine after ICI initiation (12). After the fifth cycle of cemiplimab therapy, our patient showed a TSH elevation, although without hypothyroidism symptoms, and levothyroxine therapy was increased.

Finally, our case was likely to present another irAE, namely ICI-induced hepatotoxicity (ICH). Other causes of LFTs elevation were excluded, with the limitation that specific antibodies for autoimmune hepatitis and primary biliary cholangitis were not assayed. ICH has an overall prevalence ranging between 0% and 30% (60). CTLA-4 and PD-L1 inhibitors have the highest rate of hepatoxicity, with a reported prevalence between 3%-15% and 1%-17%, respectively, while PD-1 inhibitors have a lower incidence (0%-3%) (60). ICH is usually asymptomatic and detected incidentally on routine LFTs evaluation. The onset of ICH is typically between 8 and 12 weeks after ICI start (61). A hepatocellular pattern of LFTs elevation is common, with ALT typically being higher than AST, but a cholestatic or mixed pattern of LFTs damage can be often encountered with PD-1/PD-L1 inhibitors (62). Our patient manifested an ICI-induced hepatoxicity 14 weeks after starting cemiplimab therapy with a hepatocellular pattern, and 15 weeks after restarting cemiplimab with mixed pattern. To our knowledge, few case reports of metastatic cutaneous squamous cell carcinoma (63, 64) and one case of advanced cervical cancer (65) describes hepatotoxicity secondary to cemiplimab therapy.

## 4 Conclusion

ICIs have radically improved the prognosis of cancer patients and, as a consequence, have changed the treatment landscape in the last decades. However, the immune response activation may cause specific irAEs. Endocrine irAEs are mainly thyroid dysfunctions (thyrotoxicosis or hypothyroidism) and hypophysitis, while adrenal insufficiency and DM are less common. However, DKA, a potential life-threatening condition, is frequent at diagnosis of ICI-induced DM. Therefore, physicians should be aware of ICI-induced DM; beyond the baseline assessment of glycemia and HbA_1c_ at ICI start, a close monitoring of glycemic values over time, although there is no defined timeline, is recommended during cemiplimab therapy. As DKA is a life-threatening condition, patients and their caregivers should be informed and counselled to promptly recognise DKA signs and symptoms, such as polyuria, polydipsia, weight loss, vomiting, dehydration, and change in cognitive state. If DKA develops, it is recommended to suspend ICI until glucose control is achieved with a reduction of toxicity to ≤ grade 1. The presence of positive islet autoantibodies and specific HLA susceptibility alleles may be a risk factor for developing ICI-related DKA; however, further studies are needed to clarify specific biomarkers. The development of risk-scoring tools could help to follow up more closely with those patients most at risk.

## Data Availability

The original contributions presented in the study are included in the article/supplementary material. Further inquiries can be directed to the corresponding author/s.

## Glossary

ADA: American Diabetes Association
ALT: alanine transaminase
ANA: antinuclear antibody
ASCO: American Society of Clinical Oncology
ASMA: anti-smooth muscle antibody
AST: aspartate aminotransferase
BMI: body mass index
CD8: cluster of differentiation 8
cSCC: cutaneous squamous cell carcinoma
CT: computed tomography
CTLA-4: cytotoxic T lymphocyte-associated antigen 4
DKA: diabetic ketoacidosis
DM: diabetes mellitus
EMA: European Medicines Agency
ENA: extractable nuclear antigens
ESE: European Society of Endocrinology
ESMO: European Society for Medical Oncology
FDA: Food and Drug Administration
FSH: follicle-stimulating hormone
fT3: free triiodothyronine
fT4: free thyroxine
GAD: glutamic acid decarboxylase
γGT: gamma-glutamyl transpeptidase
HbA_1c_: glycated haemoglobin
HbsAb: antibody of hepatitis B surface
HCO_3_^-^: bicarbonate
HLA: human leukocyte antigen
IA2: islet antigen 2
ICIs: immune checkpoint inhibitors
IDDM: insulin-dependent diabetes mellitus
IGF-1: insulin-like growth factor-1
IgG4: immunoglobulin G subclass 4
IFN-γ: interferon-γ
irAEs: immune-related adverse events
LFTs: liver function tests
LH: luteinizing hormone
LKM-1: liver kidney microsome type 1
MRI: magnetic resonance imaging
NCCN: National Comprehensive Cancer Network
NK: natural killer
NSCLC: non-small cell lung cancer
PaCO_2_: arterial partial pressure of carbon dioxide
PD-1: programmed cell death 1
PD-L1: programmed cell death 1 ligand
PRL: prolactin
SGLT-2: sodium-glucose co-transporter-2
SLA/LP: antibodies against soluble liver antigen/liver-pancreas
SSC: squamous cell carcinoma
T1D: type 1 diabetes mellitus
T2D: type 2 diabetes mellitus
TNF-α: tumor necrosis factor α
ZnT8: zinc transporter 8
TSH: thyroid stimulating hormone
